# Mortality prediction using SAPS II: an update for French intensive care units

**DOI:** 10.1186/cc3821

**Published:** 2005-10-06

**Authors:** Jean Roger Le Gall, Anke Neumann, François Hemery, Jean Pierre Bleriot, Jean Pierre Fulgencio, Bernard Garrigues, Christian Gouzes, Eric Lepage, Pierre Moine, Daniel Villers

**Affiliations:** 1 Professor, head of the unit of Medical intensive, Hôpital Saint Louis, Paris, France; 2 Statistician, Délégation à l'Information Médicale et Epidémiologie, AP-HP, Paris, France; 3 Statistician, center of Biostatistique Médicale, Hôpital Henri Mondor, Créteil, France; 4 Delegate to the Ministère de la Santé, Paris, France; 5 Department of Anesthésie Réanimation, Hôpital Tenon, Paris, France; 6 Professor, head of the unit of multidisciplinary internsive care, Centre hospitalier du Pays d'Aix, Aix en Provence, France; 7 Epidemiologist, Information Médicale, Hôpital de Nimes, Nimes, France; 8 Professor, Head of the Délégation à l'Information Médicale et Epidémiologie, AP-HP, Paris, and of the center of Biostatistique Médicale, Hôpital Henri Mondor, Créteil, France; 9 Department of Anesthesiology, University of Colorado Health Science Center, Denver, Colorado, USA; 10 Professor, Head of the unit of Medical intensive care, Hôpital de l'Hotel Dieu, Nantes, France

## Abstract

**Introduction:**

The standardized mortality ratio (SMR) is commonly used for benchmarking intensive care units (ICUs). Available mortality prediction models are outdated and must be adapted to current populations of interest. The objective of this study was to improve the Simplified Acute Physiology Score (SAPS) II for mortality prediction in ICUs, thereby improving SMR estimates.

**Method:**

A retrospective data base study was conducted in patients hospitalized in 106 French ICUs between 1 January 1998 and 31 December 1999. A total of 77,490 evaluable admissions were split into a training set and a validation set. Calibration and discrimination were determined for the original SAPS II, a customized SAPS II and an expanded SAPS II developed in the training set by adding six admission variables: age, sex, length of pre-ICU hospital stay, patient location before ICU, clinical category and whether drug overdose was present. The training set was used for internal validation and the validation set for external validation.

**Results:**

With the original SAPS II calibration was poor, with marked underestimation of observed mortality, whereas discrimination was good (area under the receiver operating characteristic curve 0.858). Customization improved calibration but had poor uniformity of fit; discrimination was unchanged. The expanded SAPS II exhibited good calibration, good uniformity of fit and better discrimination (area under the receiver operating characteristic curve 0.879). The SMR in the validation set was 1.007 (confidence interval 0.985–1.028). Some ICUs had better and others worse performance with the expanded SAPS II than with the customized SAPS II.

**Conclusion:**

The original SAPS II model did not perform sufficiently well to be useful for benchmarking in France. Customization improved the statistical qualities of the model but gave poor uniformity of fit. Adding simple variables to create an expanded SAPS II model led to better calibration, discrimination and uniformity of fit, producing a tool suitable for benchmarking.

## Introduction

The standardized mortality ratio (SMR) is commonly used to assess the performance of intensive care units (ICUs) by comparing the observed hospital mortality with the mortality predicted by statistical models [1,2]. This approach is valid only when it is used with models characterized by excellent calibration and discrimination [3]. Calibration reflects the agreement between individual probabilities and actual outcomes, whereas discrimination is the model's ability to separate patients who die from those who survive. Available models, such as that using the Simplified Acute Physiology Score (SAPS) II [4], are outdated [5] and must be adapted to current ICU populations [6,7].

We developed an expanded version of the SAPS II score, and we compared the performance of this new mortality prediction model with the performances of the original SAPS II and a customized SAPS II in a large population of ICU patients. Our study hypothesis was that expanding the SAPS II by adding routinely collected variables would improve mortality prediction without increasing the burden of data collection, thus producing a tool suitable for ICU benchmarking.

To expand the SAPS II, we chose variables that were easy to collect, measured on the first ICU day and routinely entered into the French national healthcare database. Furthermore, we opted not to use diagnoses; this is because ICU patients often have several diagnoses and because we wanted to develop a model suitable for evaluating ICU performance in patients with specific diagnoses. We made an exception of drug overdose because this diagnosis is common in some ICUs (up to 40% of admissions) and has a very low SMR (0.21) [8], and so a large number of drug overdose cases may result in overestimation of unit performance. In addition, the diagnosis of drug overdose is easily established at ICU admission.

## Materials and methods

We used the data entered between 1 January 1998 and 31 December 1999 into the national healthcare database, which compiles standardized data on all patients admitted to healthcare facilities in France. Among the 106 ICUs that agreed to participate (listed in the Appendix), there were 34 medical ICUs (32%), 18 surgical ICUs (17%) and 54 medical/surgical ICUs (51%). Forty-six ICUs (43%) were in teaching hospitals.

### Data collection

We developed specific software in order to extract study data from the French national healthcare database. The data entered in the database (Table 1) include the following: SAPS II score, age and sex, clinical category (medical patient or not), the patient's location before ICU admission, hospital length of stay before ICU admission, and whether the patient was admitted for a drug overdose as defined by ICD-10-CM (International Classification of Diseases, 10th revision, Clinical Modification) codes from T360 to T509.

### Mortality prediction models evaluated in the study

Three mortality prediction models were compared: the original SAPS II model, a customized SAPS II model and an expanded SAPS II model. All three models are based on SAPS II [4]. They use logistic regression, with the probability *P *of hospital mortality being calculated as follows:

*P *= exp(logit)/(1+exp [logit])

Where the logit varies with the model. In the original SAPS II model [4], the logit was chosen as:

Logit = α_0 _+ α_1 _× (SAPS II) + α_2 _× log(SAPS II + 1)

Where α_0_, α_1 _and α_2 _are the model parameters. Fitting this model to the data [4] gave the following:

Logit^(a) ^= -7.7631 + 0.0737 × (SAPS II) + 0.9971 × log(SAPS II + 1)

Customization is a simple procedure that adapts a model to specific patient populations [9]. There are two ways to customize a model. First level customization is customization of the score itself. The second level is customization of each item of the score. This latter was not performed here because it would require data that were not routinely available.

For the present study we developed a customized version of the SAPS II model for patients admitted to ICUs in France in 1998 and 1999. To this end, we used the logit of the original SAPS II model and we estimated α_0_, α_1_, and α_2 _from data from the present study.

Finally, we developed an expanded version of SAPS II by adding six variables that are potentially associated with mortality (Table 2). We transformed the continuous variables (i.e. age and hospital length of stay before ICU admission) into five-category variables. The expanded model was built using the original SAPS II approach [4]. First, we fitted a multiple logistic regression model built from the original SAPS II score and the additional variables. We used the coefficients thus obtained to define a new score, which we called the 'expanded SAPS II'. For each patient, the expanded SAPS II was the sum of the SAPS II score multiplied by the SAPS II coefficient, and the coefficients of the additional variables. Finally, we fitted a logistic regression model using the following:

Logit = β_0 _+ β_1 _× (expanded SAPS II) + β_2 _× log([expanded SAPS II] + 1)

Where β_0_, β_1 _and β_2 _are the model parameters.

### Model validation

To evaluate calibration, we measured the differences between observed and predicted mortality by using the Hosmer–Lemeshow test and by analyzing the uniformity of fit across several variables. According to the Hosmer–Lemeshow test [10], patients are first sorted by increasing mortality probability and then grouped together into 10 subgroups of patients. A low *P *value for the Hosmer–Lemeshow test indicates poor calibration across these groups. A *P *value greater than 0.1 indicates good calibration. Uniformity of fit compares observed and predicted mortality within groups of patients defined by a variable, for example patient sex or time in the hospital before ICU admission. We evaluated uniformity of fit for all variables in the expanded SAPS II (Table 2).

We evaluated discrimination based on the area under the receiver operating characteristic (ROC) curve [11]. With this method, a larger area indicates better discrimination. To compare the areas under the ROC curves for two different models calculated from the same validation set, we used the test developed by Hanley and Haijan-Tilaki [12], which is available online [13].

Because the usefulness of a mortality prediction model is largely dependent on its ability to adapt to different populations, evaluations should ideally be conducted in samples that differ from that used to develop the model. Therefore, we randomly split our data set into a training set and a validation set, both equal to half of the total sample size. We developed the mortality prediction models using the training set and then tested them using the validation set for external model validation. In addition, we used an internal validation procedure involving K-fold cross-validation on the training set itself [14]. To this end, we split the training set into K parts of similar sizes. Each part was used to validate the model fitted to the other parts (K - 1). This allowed us to evaluate not only average model performance but also performance variation due to variability in the data sets used for model fit and validation, respectively. This latter aspect of model validation is not captured when using a single data set. We used K = 5, as recommended by others [14].

### Standardized mortality ratio

The SMR is calculated as the ratio of observed hospital mortality over predicted hospital mortality, which is the sum of individual mortality probabilities. An approximate 95% confidence interval (CI) for the SMR was calculated by using the method proposed by Breslow and Day [15].

## Results

The 106 ICUs included in the study provided data for 107,652 consecutive first admissions. We successively excluded admissions with invalid SAPS II scores, burn patients, coronary patients and cardiac surgery patients, as well as those younger than 18 years. This left 77,490 (72%) patients. Among the 106 ICUs, 22 (21%) failed to provide the SAPS II score for more than 20% of admissions (some collected SAPS I rather than SAPS II). The main characteristics of the study patients are reported in Table 1. The patient mean (± standard deviation) age of the patients was 56.7 ± 18.9 years. There was a predominance of males (59%) and of medical patients (73%). Drug overdose was observed for 12% of admissions, but the range was wide, from 0% to 40% of reported cases. The mean SAPS II score was 36.1 ± 21.2. Overall ICU mortality was 18.0% and overall hospital mortality was 21.5%.

### The two mortality prediction models derived from the original SAPS II model

The customized SAPS II model was characterized by the following logit:

Logit^(b) ^= -8.1834 + 0.0467 × SAPS II + 1.3287 × log(SAPS II + 1).

The expanded model was fitted to the data, as shown in Table 2. The logit of the expanded model was as follows:

Logit^(c) ^= -14.4761 + 0.0844 × (expanded SAPS II) + 6.6158 × log(expanded SAPS II + 1).

### Validation of the three mortality prediction models

Table 3 summarizes the model validation results for all three models, and Table 4 shows their uniformity of fit across various patient subgroups.

The calibration of the original SAPS II model was poor because it strongly over-predicted mortality. SMR values exhibited wide variations across patient subgroups (Table 4); for instance, they varied from 0.62 to 0.98 across the age range, from 0.76 to 1.22 across the range of hospital lengths of stay before ICU admission, and from 0.21 to 0.90 in patients with and without drug overdose. The SMR on the validation set was 0.841 (95% CI 0.823–0.859). Discrimination, in contrast, was good, with an area under the ROC curve of 0.858 (Table 3, external validation).

With the customized SAPS II model calibration was better, with a *P *value of 0.78 by the Hosmer–Lemeshow test (Table 3, external validation). No improvement in uniformity of fit was noted as compared with the original SAPS II model, with the only exception being the clinical category. However, SMR values varied around the target value 1. The SMR on the validation set was 1.009 (95% CI 0.987–1.031). The area under the ROC curve was the same as for the original SAPS II model.

The expanded SAPS II model exhibited excellent calibration, with Hosmer–Lemeshow test *P *values of 0.81 on the validation set and 0.28 in the internal validation procedure. Uniformity of fit was clearly improved. For none of the variables included in the expanded SAPS II model was the SMR value for patient subgroups significantly different from 1. The SMR on the validation set was 1.007 (95% CI 0.985–1.028). The area under the ROC curve was 0.879 – a value significantly greater than the areas obtained with the other two models (*P *< 0.0001 using the Hanley test).

### Comparison of standardized mortality ratios across study intensive care units

**Figure 1:**
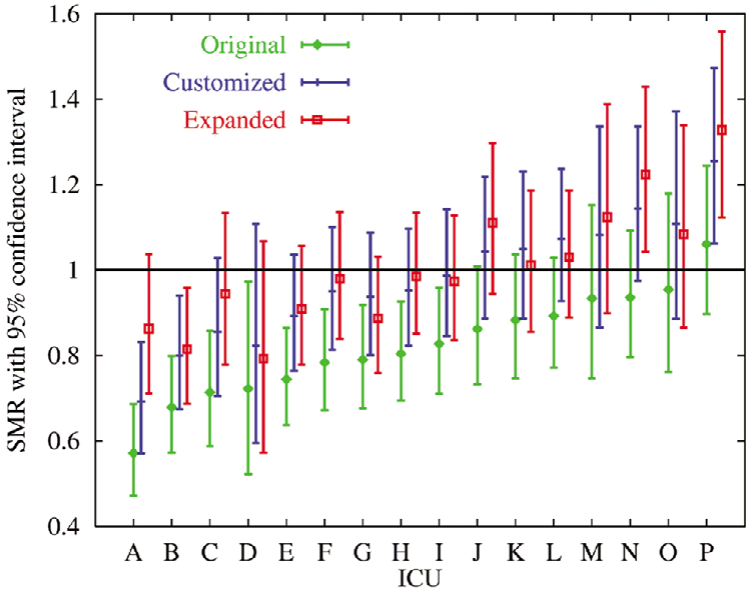
ICU performance as assessed using the three SAPS II models. Shown are standardized mortality ratio (SMR) values for 16 units with more than 300 patients, using either the original, the expanded, or the customized Simplified Acute Physiology Score (SAPS) II. A–P indicate different intensive care units (ICUs).

First, for each mortality prediction model we compared the SMRs for the 97 ICUs that contributed a sufficient number of patients. The original SAPS II model yielded SMR values between 0.40 and 1.54. Of the 97 ICUs, 43 had values smaller than 1. The SMR values given by the customized SAPS II model varied between 0.48 and 1.89; 11 units had values smaller than 1. The expanded SAPS II model produced SMR values between 0.45 and 1.67; nine units had values smaller than 1. The results for the 16 ICUs with the largest number of patients are summarized in Fig. 1.

When we evaluated differences between the customized and expanded SAPS II model, we found that seven ICUs had SMRs significantly different from 1 according to the customized SAPS II model but not according to the expanded SAPS II model (e.g. ICU A in Fig. 1). Conversely, three other ICUs had SMRs significantly different from 1 according to the expanded SAPS II model but not the customized SAPS II model (e.g. ICU N in Fig. 1).

## Discussion

Since the first reports of scoring systems for evaluating disease severity in ICU patients, many studies conducted in widely diverse ICUs and countries have highlighted the limitations of these systems for evaluating databases different from the ones in which they were developed. In addition, published scoring systems were developed many years ago (nearly 20 years for Acute Physiology and Chronic Health Evaluation [APACHE] II [16] and 10 for SAPS II [4] and APACHE III [17]).

To improve the performance of available scoring systems, two methods have been used. Customization has been investigated, for instance, by Moreno and Apolone [6] and by Metnitz and coworkers [7]. Le Gall and coworkers [9] customized SAPS II and MPM II for patients with early severe sepsis. Moreno and Apolone [6] compared two customization strategies, one using the original MPM II logit as an independent variable (first level customization) and the other using all of the original variables (second level customization). They found that second level customization was more effective in improving the overall goodness of fit of MPM II [18] and suggested that this method is preferable over first level customization. Adding variables to the scoring system is the other method used to improve performance [19].

Models that predict mortality accurately and that perform well in various ICU populations are essential to benchmarking. Glance and coworkers [5] recently investigated whether the identity of ICU quality outliers varied with the scoring system used for SMR calculation. They found that the APACHE II, SAPS II and MPM II exhibited only fair to moderate agreement in identifying quality outliers. They concluded that existing models were of limited usefulness for benchmarking.

The present study focused on SAPS II, which is routinely collected in France in all ICUs. We started the study in 2000, and so only data from 1988 and 1999 were available. Because it would have been rather difficult and time consuming to extract data from hundreds of hospitals that did not have the same software for data collection, we had to develop specific software to extract the primary data. The second version of the software was found to be efficient, the first version being bugged and not allowing proper analysis. Since 2003 there has been a national database, and we are now able to benchmark units easily using expanded SAPS II. Nevertheless, we must always seek prior permission from the units to analyze their data anonymously. On the other hand, SAPS III is now published [20,21], and one of the authors of the present report (JRLG) participated in the creation of SAPS III. The SAPS III appears very promising, and is more recent and sophisticated than SAPS II. Nevertheless, for historical comparisons the expanded SAPS II can easily be obtained from existing databases.

We expanded SAPS II by adding other robust and simple data that are routinely available. Apart from drug overdose, we did not include diagnoses in the model because ICU benchmarking for a specific diagnosis (such as acute respiratory distress syndrome, severe pancreatitis, peritonitis, chronic obstructive pulmonary disease, or pneumonitis) cannot be achieved using a model in which that diagnosis is included. Nevertheless, we made an exception for drug overdose for several reasons. First, drug overdose is a simple diagnosis that is established on the first ICU day; although data collection in the French national healthcare database allows reporting of drug overdose at any time during the ICU stay, in practice ICU patients with drug overdose are admitted for this reason. Second, the percentage of patients with drug overdose was high in the overall database (12%) but varied widely across ICUs (from 0% to 40%). Finally, the SMR for drug overdose is very low (0.21 with the original SAPS II and 0.25 with the customized SAPS II), which may artificially improve the SMR in ICUs with large numbers of drug overdose cases. Introducing drug overdose into the model gives a mean SMR close to 1 for this diagnosis.

The exclusive use of data entered into the French healthcare database allowed us to include a large number of ICU stays and to develop a mortality prediction model suitable for benchmarking, without additional data collection. To benefit from these fundamental advantages, we did not use second level customization of the SAPS II because the components for this procedure are not routinely available. Also, we did not include organ failures because their timing is not routinely recorded in the database.

All three SAPS II models produced fairly satisfactory areas under the ROC curve (range 0.858–0.879). Nevertheless, discrimination was significantly better with the expanded SAPS II model than with the other two models. There were much greater differences in calibration across the three models. The original SAPS II model markedly overestimated mortality and yielded poor uniformity of fit. Customization improved calibration, yielding a *P *value of 0.78 using the Hosmer–Lemeshow test, but it did not improve uniformity of fit. Good uniformity of fit was obtained using the expanded SAPS II model. With this model, the SMR values for patient subgroups were not significantly different from 1.

In the present study, the expanded SAPS II model performed much better than the original SAPS II model and significantly better than the customized SAPS II model, in particular in terms of uniformity of fit. All required variables are collected consistently over time and across ICUs, and can be extracted from existing databases using dedicated abstracting software.

Comparisons across ICUs of SMRs obtained using the three models revealed large differences. With the original SAPS II model, 43 of 97 ICUs (44%) had SMR values significantly smaller than 1. Because the original model cannot be used for benchmarking, we focused our comparison on the customized and expanded models. SMRs ranged from 0.48 to 1.89 with the customized model and from 0.45 to 1.67 with the expanded model. There were 10 ICUs with SMR values significantly different from 1 with the customized SAPS II model but not with the expanded SAPS II model or *vice versa*, indicating that use of a customized model for benchmarking might be misleading.

In our study, the quality of data was not perfect. Data were not collected specifically for the study but were taken from standardized reports. The completeness of data in the reports was evaluated elsewhere [22], with special attention given to SAPS II score. The SAPS II score was reported for 80% of stays. This is because some administrative units included intermediate units that only monitored patients [22]. In these patients collection of the SAPS II score is not mandatory. In addition, a formal quality control analysis of the French database has been published [23], showing mainly that there was an underestimation of comorbid conditions, which are not part of the expanded SAPS II. Strengths of our study include the large number of patients and the use of a real-life data source.

## Conclusion

The original SAPS II model is not suitable for ICU benchmarking, and neither is customization of the SAPS II entirely satisfactory. We were unable to customize its components, which probably would have been more satisfactory. The expanded SAPS II model obtained by adding simple data that are routinely recorded in French national healthcare database may be a good compromise between immediate, nationwide applicability and adequate model performance. Discrimination, calibration and uniformity of fit – three properties that we believe are essential for benchmarking – were far better with the expanded SAPS II model. For some units, the expanded SAPS II model exhibited good or poor performance not detected by the customized SAPS II model.

Although SMR is one aspect of an ICU's performance, we must remind practitioners and administrative managers that there are other aspects of performance, namely patient and family satisfaction, nurse turnover and burnout, costs and organizational issues.

## Key messages

• 	The original SAPS II mortality prediction model is outdated and must be adapted to current ICU populations.

• 	The original SAPS II may be used to score severity of illness in ICU patients, but it is necessary to use the expanded SAPS II to calculate the SMR or to measure the performance of ICUs.

• 	Adding simple data routinely collected to the original SAPS II led to better calibration, discrimination and uniformity of fit of the model.

• 	The stastistical qualities of the expanded SAPS II are much better than those of the original and the customized SAPS II.

• 	Above all, the expanded SAPS II is easy to obtain from the existing databases. It is a simple system that may be used to measure precisely the performance of units and to compare performance over time.

## Abbreviations

APACHE = Acute Physiology and Chronic Health Evaluation; CI = confidence interval; ICU = intensive care unit; MPM = Mortality Probability Model; ROC = receiver operating characteristic; SAPS = Simplified Acute Physiology Score; SMR = standardized mortality ratio.

## Competing interests

The authors declare that they have no competing interests.

## Authors' contributions

JR conducted the study and drafted the manuscript. AN performed statistical analysis. FH collected and managed the data. JPB, JPF, BG, CG, EL, PM and DV conceived the study in terms of its design and coordination, and participated in data analysis. DV also conducted the study and helped to draft the manuscript. All authors read and approved the final manuscript.

## Figures and Tables

**Table 1:** Demographic data

Characteristics	All patients (n = 77,490)	Training set (n = 38,745)	Validation set (n = 38,745)	P
Age (years; mean ± standard deviation)	56.71 ± 18.91	56.70 ± 19.00	56.72 ± 18.83	0.9422

Age (%)				
<40 years	22.20	22.35	22.05	
40–59 years	27.95	27.77	28.13	
60–69 years	18.47	18.40	18.54	
70–79 years	21.68	21.65	21.71	
>79 years	9.69	9.82	9.57	
Men (%)	59.31	59.06	59.56	0.1581
Medical patients (%)	73.49	73.46	73.53	0.8261
Patient origin (%)				
Emergency room or mobile emergency unit	49.94	49.75	50.13	0.1345
Ward in same hospital	39.91	39.89	39.94	
Other hospital	10.15	10.36	9.93	
				
Length of hospital stay before ICU admission (%)				
<24 hours	67.75	67.76	67.73	
1 day	12.37	12.56	12.18	
2 days	4.67	4.57	4.77	
3–9 days	9.5	9.59	9.42	
>9 days	5.71	5.52	5.9	
Medicine overdose (%)	11.86	11.94	11.79	0.5122
Original SAPS II score				
Maximum	162.00	159.00	162.00	0.5817
Median	32.00	32.00	32.00	
Minimum	1	1	1	
ICU mortality (%)	17.99	17.95	18.03	0.7647
Hospital mortality (%)	21.48	21.39	21.58	0.5289

*P *value obtained by the Wilcoxon test for quantitative variables and the χ^2 ^test for qualitative variables. ICU, intensive care unit; SAPS, Simplified Acute Physiology Score.

**Table 2:** Expanded SAPS II scoring system sheet

Variable	Points
Original SAPS II score	0.0742 × SAPS II
Age	
<40 years	0
40–59 years	0.1639
60–69 years	0.2739
70–79 years	0.3690
>79 years	0.6645
Sex	
Male	0.2083
Female	0
Length of hospital stay before ICU admission	
<24 hours	0
1 day	0.0986
2 days	0.1944
3–9 days	0.5284
>9 days	0.9323
Patient's location before ICU	
Emergency room or mobile emergency unit	0
Ward in same hospital	0.2606
Other hospital	0.3381
Clinical category	
Medical patient	0.6555
Other	0
Intoxication	
No	1.6693
Yes	0

Logit = -14.4761 + 0.0844 × score + 6.6158 × log(score + 1). The expanded Simplified Acute Physiology Score (SAPS) II score is the sum of the points for a given patient. ICU, intensive care unit.

**Table 3:** Calibration and discrimination of the models

Model	Internal validation (fivefold cross-validation on the training set)				External validation on the validation set		
	
	*P *value of Hosmer–Lemeshow test		Area under the ROC curve		Hosmer–Lemeshow test		Area under the ROC curve
	
	Mean	Standard deviation	Mean	Standard deviation	Test statistic C	*P *value	
Original SAPS II	0.001	0.001	0.8591	0.0058	1162.9	<0.0001	0.8575
Customized SAPS II	0.6280	0.1422	0.8562	0.0058	6.41	0.7794	0.8575
Expanded SAPS II	0.2754	0.3832	0.8797	0.0054	6.04	0.8116	0.8787

ROC, receiver operating characteristic; SAPS, Simplified Acute Physiology Score.

**Table 4:** Uniformity of fit of the three SAPS II models in the validation sample

Variable	Value	SMR		
		
		Original	Customized	Expanded
Age				
	<40 years	0.62*	0.74*	1.05
	40–59 years	0.77*	0.92*	1.02
	60–69 years	0.86*	1.03	1.00
	70–79 years	0.90*	1.09*	1.00
	>79 years	0.98	1.18*	0.99
Sex				
	Male	0.88*	1.05*	1.00
	Female	0.79*	0.95*	1.02
Length of inhospital stay before ICU admission				
	<24 hours	0.76*	0.91*	1.00
	1 day	0.89*	1.05	1.04
	2 days	0.93	1.12*	1.02
	3–9 days	1.07*	1.28*	1.01
	>9 days	1.22*	1.46*	1.01
Patient's location before ICU				
	From outside	0.71*	0.86*	1.00
	From the wards	0.99	1.19*	1.02
	From another hospital	0.89*	1.07*	0.99
Clinical category				
	Medical patient	0.85*	1.02	1.04
	Other	0.80*	0.96	1.00
Intoxication				
	No	0.90*	1.08*	1.01
	Yes	0.21*	0.26*	1.04

*The 95% confidence interval does not include 1. ICU, intensive care unit; SAPS, Simplified Acute Physiology Score.
